# A Forward GPS Multipath Simulator Based on the Vegetation Radiative Transfer Equation Model

**DOI:** 10.3390/s17061291

**Published:** 2017-06-05

**Authors:** Xuerui Wu, Shuanggen Jin, Junming Xia

**Affiliations:** 1 Shanghai Astronomical Observatory, Chinese Academy of Sciences, Shanghai 200030, China; sgjin@shao.ac.cn; 2 Key Laboratory of Planetary Sciences, Chinese Academy of Sciences, Shanghai 200030, China; 3 National Space Science Centers, Chinese Academic of Sciences, Beijing 100190, China; Xiajunming@nssc.ac.cn

**Keywords:** GNSS-R, multipath, radiative transfer equation model, vegetation, simulation

## Abstract

Global Navigation Satellite Systems (GNSS) have been widely used in navigation, positioning and timing. Nowadays, the multipath errors may be re-utilized for the remote sensing of geophysical parameters (soil moisture, vegetation and snow depth), i.e., GPS-Multipath Reflectometry (GPS-MR). However, bistatic scattering properties and the relation between GPS observables and geophysical parameters are not clear, e.g., vegetation. In this paper, a new element on bistatic scattering properties of vegetation is incorporated into the traditional GPS-MR model. This new element is the first-order radiative transfer equation model. The new forward GPS multipath simulator is able to explicitly link the vegetation parameters with GPS multipath observables (signal-to-noise-ratio (SNR), code pseudorange and carrier phase observables). The trunk layer and its corresponding scattering mechanisms are ignored since GPS-MR is not suitable for high forest monitoring due to the coherence of direct and reflected signals. Based on this new model, the developed simulator can present how the GPS signals (L1 and L2 carrier frequencies, C/A, P(Y) and L2C modulations) are transmitted (scattered and absorbed) through vegetation medium and received by GPS receivers. Simulation results show that the wheat will decrease the amplitudes of GPS multipath observables (SNR, phase and code), if we increase the vegetation moisture contents or the scatters sizes (stem or leaf). Although the Specular-Ground component dominates the total specular scattering, vegetation covered ground soil moisture has almost no effects on the final multipath signatures. Our simulated results are consistent with previous results for environmental parameter detections by GPS-MR.

## 1. Introduction

Global Navigation Satellite Systems (GNSS) have reached a new era with wider applications than navigation, timing and positioning, e.g., GNSS-Reflectometry (GNSS-R). Compared to traditional remote sensing techniques, GNSS-R has advantages of exploiting pre-existing transmission sources with wide spreading applications from meso-scale ocean remote sensing to soil moisture and vegetation detections on the land surface [1].

In order to receive the reflected signals, a special receiver should be designed, such as the modified DMR (Delay Doppler Maps Receiver) used in SMEX (Soil Moisture Experiment) airborne GPS-R remote sensing experiments [2]; and BAO-Tower (Boulder Atmospheric Observatory-tower) experiments [3] or the SAM sensor (An innovative GNSS-R system for Soil Moisture retrieval) used in the LEiMON (Land Monitoring with Navigation Signals) experiments [4]. During the LEiMON project, a GNSS-R simulator for bare and vegetated soils was developed [5]. Egido et al. has also carried out experimental activities for soil moisture and vegetation biomass study [6,7].

As for GPS-Interferometric Reflectometry (GPS-IR), an efficient method for geophysical parameter retrieval is to employ the interferometric signals of direct signals and reflected signals. A specially designed GPS receiver named SMIGOL (Soil Moisture Interference pattern GNSS Observations at L-band Reflectometer) [8] or an extension of PSMIGOL (dual-polarization SMIGOL) has been designed [9]. Based on the in-situ measurements, Rodriguez-Alvarez et al. have given good quantitative retrieval results.

In addition, a widely used geodetic GPS receiver can also be used to remotely sense the near-surface soil moisture, vegetation, as well as snow depth using a technique known as GPS multipath reflectometry (GPS-MR). Three GPS interferogram metrics extracted from the GPS multipath observables, namely, effective reflector height, phase and amplitude. GPS sites data from PBO (plate boundary observatory) have been used in the study of soil moisture where it was found that phase was linearly correlated with surface soil moisture, while the other two metrics (effective reflector height and amplitude) had nonlinear relationships with soil moisture [10]. Experimental data from PBO and SNOTEL (Snow Telemetry) showed that effective reflector height was an efficient metric for snow depth retrieval (the correlation parameter is 0.7~0.9) [11]. Similarly Chew et al. pointed out that when the vegetation wet weight was below 1.5 kg·m^2^, the metric of amplitude could be used to estimate vegetation amount. Since phase was very sensitive to soil moisture [10], it was not a suitable indicator for detecting vegetation changes [12].

A 1-D plane-stratified model has previously been employed in their vegetation amount study [12]. The model is separated into two stages, with the first being the permittivity profile generation that is then input into the second stage (reflector power at the antenna). The intention of the 1-D plane-stratified model was to divide the soil depth and vegetation canopy into a 1-D stratified permittivity profile, which input into the right- and left-handed reflection coefficients. The reflection coefficients were then combined with the corresponding antenna gain to get the final reflected power at the antenna.

To better understand the internal mechanisms, it is necessary to develop an appropriate multipath model. Currently there are three kinds of GPS multipath simulators: (1) a tracking simulator assuming arbitrary values for the reflected power; (2) a geometry simulator adopting empirical values, and (3) a polarimetric model that calculates the complex reflectivity. However, most of the present models are not available to users. Recently, Nievinski and Larson [13,14] developed a forward GPS multipath simulator that was based on the physical model proposed by Zavorotny et al. [15]. This model combined the antenna type and the surface characteristics.

Microwave scattering models are used to describe the scattering properties at the microwave bands, which can be either emissivity model (radiometer) or backscattering models (monostatic radar), but as for the GNSS-R technique, the microwave scattering model of bistatic scattering model should be used. That is, microwave scattering models include bistatic scattering model. As for the techniques used to solve the microwave scattering problems, most of the models are based on the distorted Born approximation (DBA) theory or radiative transfer (RT) theory, while, in this paper, a vegetation bistatic scattering model based on RT theory is employed, i.e., Bistatic-Michigan Microwave Canopy Scattering Model [16,17]. In order to make it suitable for GPS-MR, only specular scattering is considered. One limitation of using GPS-MR measurements is that the model is not suitable for forest regions since the GPS sites are rarely located in forests due to obscuration of direct GPS signals [18]. However, the technique is suitable for the majority of vegetation types, such as cropland, grassland and shrubland. Therefore, in this paper, we ignore the trunk layer and its corresponding scattering mechanisms, considering only the crown layer and ground layer that remain when simulating the relatively low agriculture (compare to the high forest).

After incorporated the Bi-mimics model into the forward GPS multipath simulator [13,14,16], a new simulator is formed, which is the first time for a combination of a forward GPS multipath simulator with vegetation radiative transfer equation model. Using our new developed GPS multipath model, the interactions of GPS signals (L1, L2, C/A, P(Y) and L2C modulations) with vegetated targets can be better understood, which will be helpful in data explanation, field experiment design, and vegetation parameter retrieval from GPS-MR. In next sections, we present the theory and methodology used in this study as well as the simulation results.

## 2. Theory and Methodology

**Figure 1:**
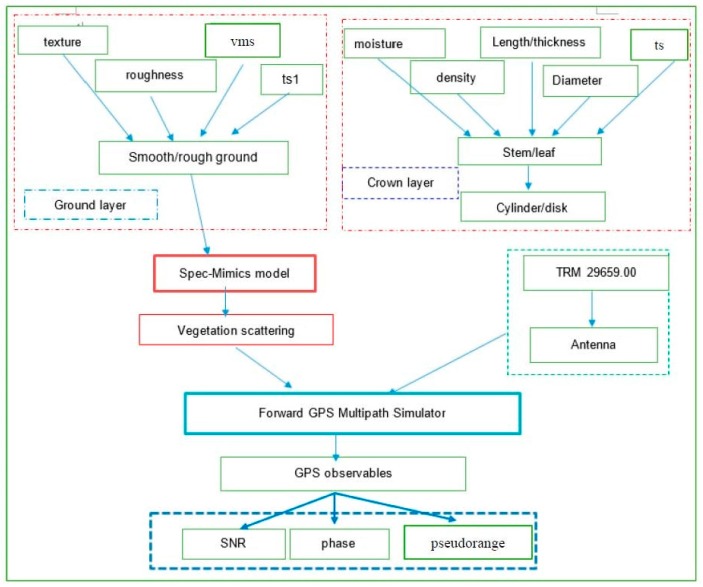
Flowchart of the improved forward GPS multipath simulator. The forward GPS multipath simulator (in the green box) is mainly composed of the effects of vegetation scattering and antenna type. The main improvement of the simulator is to use the Spec-Mimics model (in the red box) to represent the vegetation scattering. TRM 29659.00 is a representative of the antenna type (in the green dotted lines). Those parts in the red dotted lines are the frame of the Spec-Mimics model: ground layer and crown layer. The parameters in the red dotted line boxes are the model inputs, while the ones in the blue box are outputs of the model. Soil texture is represented by the percentages of sand and clay in soil, respectively, its roughness is characterized by rms height and correlation length. Vms is the volumetric soil moisture content, ts1 is the temperature of the soil, as for the crown layer, stem or leaf are represented by the dielectric scatters of cylinders or disks, respectively. Moisture content, density, length/thickness, diameter and temperature of the scatters are the inputs for the crown layer. SNR, phase and pseudorange are the final outputs of the simulator.

The aim of the newly developed GPS multipath simulator is to focus on the physical scattering mechanism of the reflected surface. The commonly used Bi-Mimics model based on the RT theory is employed to describe the scattering properties of the reflected surface. The forward GPS multipath simulator has been developed by combining the antenna type with microwave radiative transfer equation model. The simulator flowchart is presented in Figure 1. Model inputs for the soil layer include soil texture, surface roughness (in the presence of RMS height, surface correlation length and surface correlation function), soil moisture (in the presence of volumetric soil moisture) and soil temperature. The vegetation layers are modeled as different scatters, and their simulator inputs include the vegetation moisture content, scatter density, scatter length, scatter thickness, scatter diameter and vegetation temperature. Vegetation input parameters are given in Table 1. A Trimble choke ring antenna has been selected as the default antenna type since the objective of the study is not to consider the effects of various antenna types on GPS multipath observables, but to take the effects of scattering surface parameters into account. Signal-to Noise Ratio (SNR), carrier phase multipath error and pseudorange code multipath error are the model outputs, where errors are the difference with respect to multipath-free outputs.

### 2.1. Radiative Transfer Theory

**Figure 2:**
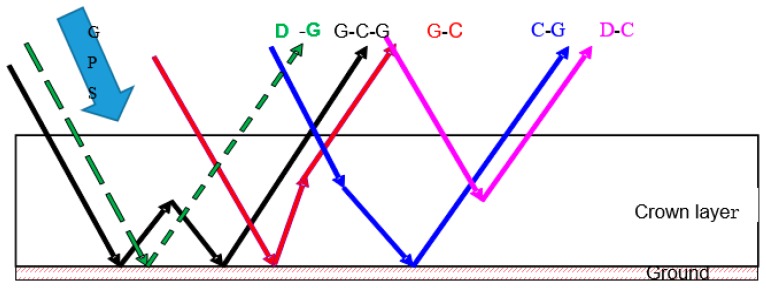
Scattering mechanisms in the Bi-Mimics model (only the crown layer and the ground layer are retained, while the trunk layer has been eliminated) including D-G (direct ground), G-C-G (ground reflection and crown scattering and ground reflection), G-C (ground reflection and crown scattering), C-G (crown scattering and ground reflection) and D-C (direct crown scattering). The specular ground reflection is not shown in the figure (S-G). For the same scattering mechanism, it has the same color for the arrows and the top legend.

Mimics (Michigan Microwave Canopy Scattering Model) model has been developed for monostatic (backscattering) radar systems [17]. However, GNSS-R is typically a bistatic radar, so a bistatic scattering model referred to as Bi-Mimics model has been developed based on Liang and coworkers’ model [16]. The Bi-Mimics model is based on the first-order solution of RT model, where first-order refers to solving with a single scattering from each region and double scattering from pairs of regions. This model comprises a crown layer, a trunk layer and a rough-surface ground boundary. Dielectric cylinders (representing needles and branches) and disks (representing leaves) are used to model the crown layer. The trunk layer is simulated by large vertical dielectric cylinders of uniform diameter. The underlying ground is modeled as a rough dielectric surface using a root mean square (RMS) height and a correlation length to characterize its roughness properties. The Bistatic scattering model based on RT theory is used to describe the changes of propagating GPS intensity by the process of extinctions and emissions. Due to the coherence limitations of direct and reflected signals of GPS-MR and the obscuration of the direct signals, this technique is not suitable for large forests [18]. For this reason, only low crop or shrubs are considered and we therefore eliminate the trunk layer. The crown layer and ground layer have been reserved. The scattering mechanisms measured in bistatic directions are shown in Figure 2.

(1) $$I_{s}\left(\theta_{s},\varphi_{s}\right)=T\left(\theta_{s},\varphi_{s}\right)I_{i}\left(\theta_{i},\varphi_{i}\right)$$

Assume that the incident intensity $I_{i}$ impinges on the top surface of the canopy from the direction $\left(\theta_{i},\varphi_{i}\right)$, while the upward scattering intensity $I_{s}$ is in the direction $\left(\theta_{s},\varphi_{s}\right)$. The first-order bistatic transformation matrix T links the changes between $I_{s}$ and $I_{i}$ (1).

(2) $$\sigma_{rt}\left(\psi_{r},\chi_{r},\psi_{t},\chi_{t}\right)=4\pi {\tilde{Y}}_{m}^{r}M_{m}Y_{m}^{t}$$

Using the wave synthesis technique (Equation (2)) [19], the scattering cross sections of any combinations of transmitted and received polarizations can be got. $Y_{m}^{t}$ and $Y_{m}^{r}$ are the modified stokes vectors of the transmitted and received waves. The modified Mueller matrix $M_{m}$ is got from the transformation matrix T (Equation (1)).

As for the GPS multipath model, at present, it is thought the signals are collected from the first Fresnel Zone and therefore we need a specular scattering model. If we set the observatory angles as $\theta_{s}=\theta_{i},\varphi_{s}=\varphi_{i}$ and modify the corresponding phase, extinction and ground surface matrices, the bistatic scattering model Bi-Mimics can be applied to specular reflections is referred to as the Spec-Mimics model.

### 2.2. The Improved Multipath GPS Simulator

Recently, a fully polarimetric GPS multipath simulator based on the model developed by Zavorotny et al. has been made available to the public [13,14,15]. The model is capable of predicting GPS multipath observables (SNR, carrier phase and code pseudorange) by coupling different surfaces and antenna types. The simplified expressions of the simulator can be written as the following,

(3) $$P_{d}=P_{d}^{R}G_{d}^{R}W_{d}^{2}$$

(4) $$P_{r}=P_{d}^{R}{\left|XSW_{r}\right|}^{2}$$

where P is the electric field, *G* is the antenna gain, subscripts *d* and *r* represent the direct and reflected components, respectively, superscript *R* is the RHCP polarization, *W* is the Woodward ambiguity function and *X* is the coupled surface/antenna coefficient.

Chew et al. adapted an electrodynamics forward model to model SNR data [12]. In order to simulate the vegetation scattering, a plane-stratified model was employed. This model is mainly based on pure mathematical models regardless the nature of reflectors. Using the dielectric constant ε of different mediums (such as vegetation), we can get the corresponding Fresnel reflectivity at different incident angles θ. Although polarization and coherence are taken into account, the ground reflectivity is calculated using the Fresnel theory by different dielectric constants, such as snow, bare soil and vegetation. However, this simple form of reflectivity did not take internal canopy geometry into account and was not sufficient to describe the propagation of microwave wave (L band) in the vegetation. If we want to pay more attention to the effects of environmental parameters on the final GPS multipath observables (SNR, phase and code pseudorange), we need a bistatic scattering model to better understand the interactions of GPS signals with vegetated targets, which assist in vegetation parameter retrieval from GPS-MR measurements.

The forward GPS multipath simulator accounts for right- and left-handed circularly polarized components of the GPS broadcast signal and of the antenna and surface responses as well. Since for the improvement, we focus on the surface response, the RT model is embedded in the forward GPS multipath model. The surface coefficients part in Equation (4) is replaced with RT model (Equations (1) and (2)). It can represent the scattering mechanisms, as shown in Figure 2, while the GPS broadcast signal and antenna parts remain unchanged. This model directly links the vegetation properties with the GPS multipath observables.

## 3. Model Validation

The original GPS multipath simulator is the physical model developed by Zavorotnay et al. [15], which is an electrodynamic model of GPS direct and reflected signal interference. The model has been validated by soil moisture field experiment. The forward GPS multipath simulator developed by Nievinski and Larson [13,14] is based on the model developed by Zavorotny et al. [15], which accounts for right- and left-handed circularly polarized components of the GPS broadcast signal and the antenna as well as surface responses. For both models, they are based on pure mathematical models regardless the nature of reflectors.

For our developed model, we have utilized the right- and left-handed circularly polarized components of the GPS broadcast signal and of the antenna responses developed by models [13,14,15]. However, we modified the surface response component where the radiative transfer model (bistatic-Mimics) is employed. Equations (3) and (4) have shown the simplified expressions of the forward GPS multipath simulator [13,14]. From the equations, it can be seen that if we want to modify the surface responses, what we should do is to adapt X, which is coupled surface/antenna coefficients. As for the calculations, it needs the surface coefficients of circular polarizations (RR and LR) and linear polarizations (H and V). The model is combined by two modules, one is the RHCP and LHCP polarizations of GPS broadcast signals and antenna responses that is written by MATLAB. Since this part has already validated [13,14,15], it is feasible. The other one is the surface responses, while this part is written by Fortran. We made an interface between these modules. That is to say, using the Fortran outputs (surface response), we made an input interface for Matlab (GPS broadcast signals and antenna responses). At first, we just use the outputs of original Fresnel Reflectivity models as the interface inputs. We find the consistent results for the original forward GPS multipath simulator [13,14], namely there is no problem for the interface.

Then we need to validate the surface response part, which employs the radiative transfer model. As for the RT model, bistatic-Mimics model is used in our manuscript. Mimics model is a commonly used and validated model, but it has been developed for monostatic radar systems (backscattering) and is insufficient for GNSS-R scattering study, which needs a bistatic scattering model. According to Liang et al. [16], we modify Mimics to the bisatic form, i.e., the model can get the BRCS of vegetation at any azimuth and zenith angels. We set the angels at backscattering geometry and get the consistent scattering results with the Mimics model. In this way, we think there is no problem for the bistatic-Mimics. Since in the calculations of the forward GPS multipath simulator, we need the circular polarization scattering coefficients, we have to adapt Bistatic-Mimics polarizations. Wave synthesis technique is used to get polarizations at any combinations. By changing the modified Stokes vectors, we can get the scattering coefficients at linear polarization and circular polarizations. We set the modified stokes vector (orientation and ellipticity angles) at the linear polarizations, and find it is the same with the original Bi-Mimics model. By this way, we validate the polarization model modification. Therefore, the surface response part of the GPS multipath simulator is validated by validating the correctness of scattering geometry and polarization.

## 4. Simulations and Results 

With the above-improved GPS multipath simulator, theoretical simulations of vegetation parameters effects now provided. We begin with the comparisons between bare soil and vegetation on GPS multipath observables, where the main features of vegetation moisture content and sizes are also illustrated. Meanwhile, specular scattering cross sections calculated by the Bi-Mimics model are also presented in order to interpret the effects of vegetation characteristics on final GPS multipath observables.

### 4.1. Bare Soil and Wheat Comparisons

Models given in [20,21] are used for the calculations of soil permittivity. As for the RT model, all vegetation components are treated like different combinations of single microwave scatters: flat circular disks, dielectric cylinders or probate spheroids. Wheat is selected as a representational vegetation for simulations: its stem and leaf are modeled as dielectric cylinder and disks, respectively. Model inputs are given in Table 1.

**Figure 3:**
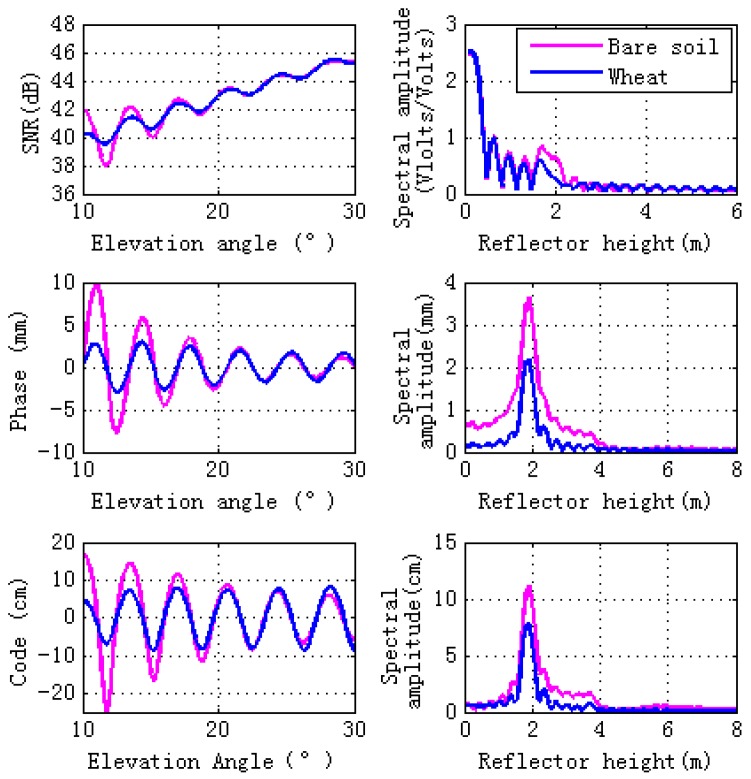
Comparisons of GPS multipath simulation between vegetation (veg) and bare soil (soil). Model inputs for bare soil are the same as the ground layer of vegetation. Bare soil is shown pink, wheat in blue. Lomb-Sargle periodograms computed for the GPS multipath signatures are shown in the right panel in plots.

Since soil texture has almost no effects on GPS multipath observables [10], sandy loam is used in our simulation. Figure 3 shows simulations for GPS L2 signal observables: SNR, carrier phase multipath error, and pseudorange code multipath error. Magnitudes of simulations, both bare soil and wheat, are consistent with the filed GPS multipath study [12] and there is a sinusoidal-like style for the final interference pattern. As can be seen from Figure 3, when the elevation angles are larger than 10° and lower than 30°, wheat decreases the magnitudes of the GPS multipath observables (SNR, phase and pseudorange). Lomb-Scargle periodograms are computed for GPS multipath signatures. The right panel of Figure 3 shows that wheat causes the peak amplitude of the GPS multipath observables to decrease, especially the phase and code pseudorange spectral amplitude.

**Figure 4:**
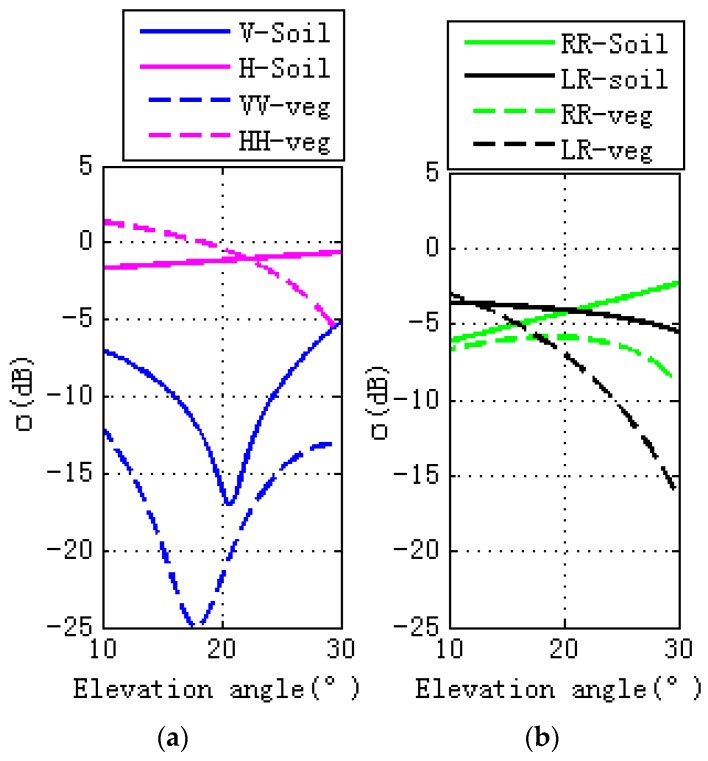
Specular scattering comparisons of vegetation and bare soil, (**a**) is for linear polarizations, while (**b**) is for circular polarizations. Bare soil and vegetation are shown, respectively, in solid and dashed line styles. VV pol is shown in blue, HH pol in pink

The differences caused by the GPS multipath observables, as shown in Figure 3, are due to the scattering surface’s properties. Specular scattering comparisons with vegetation and bare soil are shown in Figure 4, where the left figure is for linear polarizations and the right is for circular polarization. For soil and vegetation, there is a notch for V polarization in the vicinity of Brewster angle, with the scattering cross section for V polarization being larger than H polarization. For V polarization, the scattering cross section of soil is larger than vegetation. For H polarization, scattering cross section of vegetation is larger than soil when the elevation angle is between 10° and 22°. As the elevation angle varies from 22° to 30°, the scattering cross section of vegetation is larger than soil. While the right figure is for circular polarization, as for RR polarization, scattering cross section of soil is larger than vegetation, soil scattering increase with the elevation angle, while the vegetation scattering increase with the elevation angle and then decrease (from 22° to 30°). For LR polarization, when the elevation angle is between 10° and 12°, scattering cross section of vegetation is larger than soil, while for the other range of the elevation angles between 12° and 30°, the scattering cross section of the soil is larger than that of the vegetation. For both soil and vegetation, scattering cross section decreases as the elevation angle increases.

### 4.2. Wheat Moisture Effects

The effects of stem and leaf moisture contents on GPS multipath observables are investigated in this section. To demonstrate different wheat moisture content, we consider Veg 1 and Veg 2 for comparison (Table 2), with Veg 1 having a moisture content a factor of four times higher than Veg 2.

**Figure 5:**
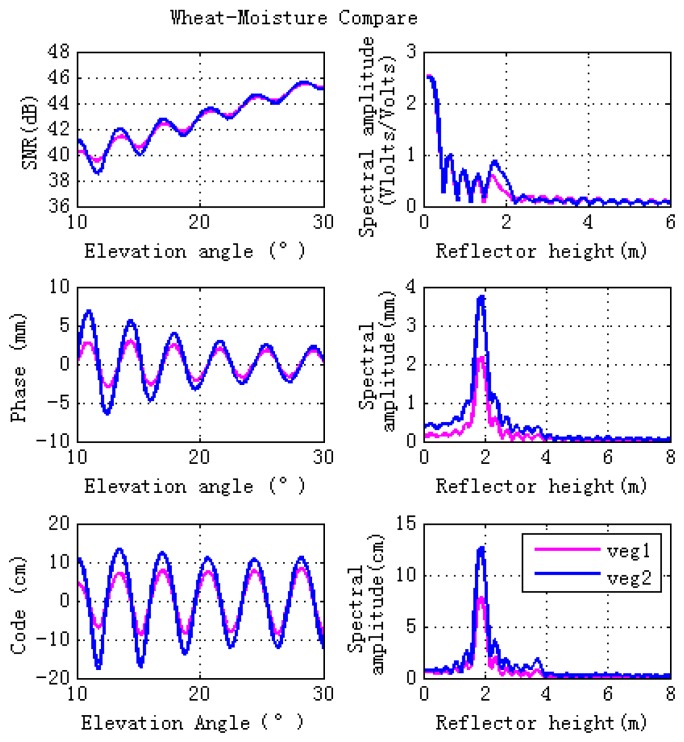
Effects of different vegetation moisture content on the forward GPS multipath simulation. The vegetation moisture content of Veg 1 (Vegetation 1) is higher than that one of Veg 2 (Vegetation 2). The moisture content (gravimetric) of stem and leaf for Veg 1 is 0.72 and 0.8, respectively, while the equipment for Veg 2 is 0.2 and 0.2, respectively. Veg 1 is shown in pink, Veg 2 in blue. Lomb-Sargle periodograms computed for the GPS multipath signatures are shown in the right hand panel.

**Figure 6:**
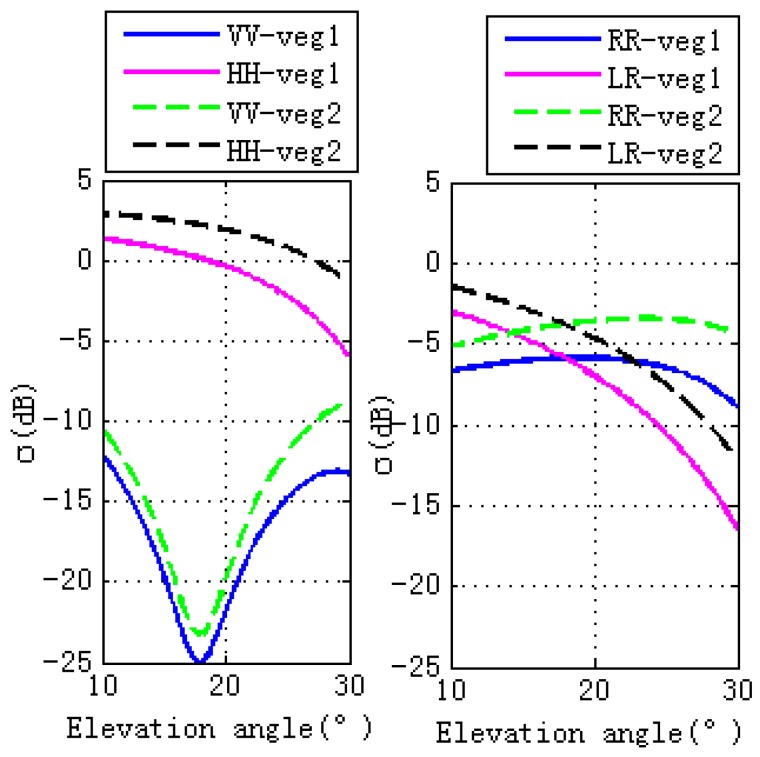
Specular scattering cross sections of different vegetation moisture contents. The left figure is for linear polarizations, while the right one is for circular polarizations. The vegetation moisture contents of Veg 1 (Vegetation 1) is larger than the one of Veg 2 (Vegetation 2). The moisture contents (gravimetric) of stem and leaf for Veg 1 are 0.72 and 0.8, respectively. While the ones for Veg 2 are 0.2 and 0.2, respectively.

Figure 5 shows the final simulations. It can be seen that higher vegetation moisture content corresponds to lower magnitude fluctuations of GPS observables, which is due to the lower specular scattering cross sections at different polarizations for the entire set of elevation angles, as shown in Figure 6. The right hand panel of Figure 5 shows that lower vegetation moisture content corresponds to higher peak amplitude of spectral amplitude (especially for phase and code pseudorange). This means that changes of vegetation moisture content affects GPS observables, indicating that GPS-MR is an efficient technique for vegetation moisture content detections, and will be an effective supplement to the existing remote sensing techniques.

### 4.3. Vegetation Height Effects

**Figure 7:**
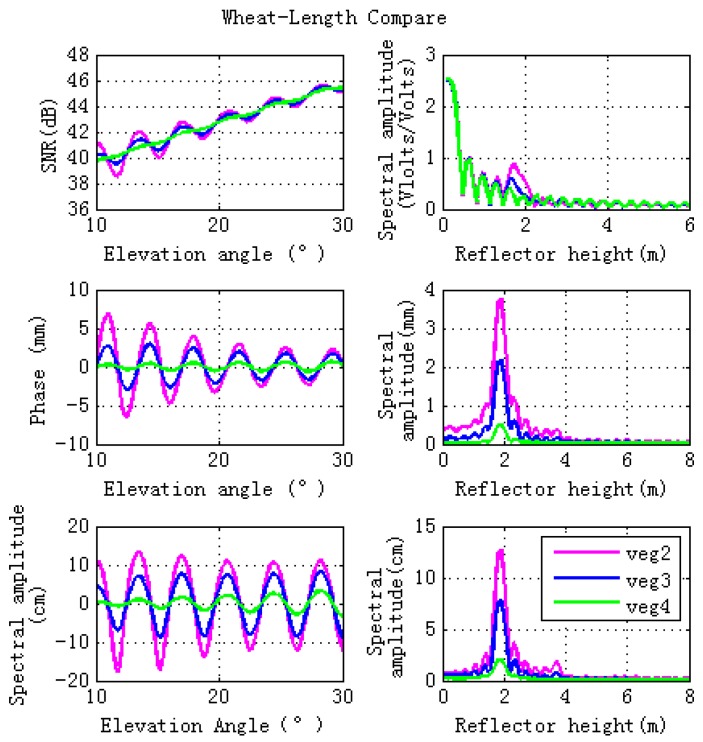
Effects of different vegetation sizes on the forward GPS multipath simulation observables. The lengths and diameters of the stems or the thicknesses and diameters of the leaves for Vegetation 2 (veg 2), Vegetation 3 (veg 3) and Vegetation 4 (veg 4) are different. The sizes of veg 2 are smaller than veg3, while the sizes of veg 4 are largest. Results for varying wheat lengths are shown in pink (veg 2), blue (veg 3) and green (veg 4). Lomb-Sargle periodograms computed for the GPS multipath signatures are shown in the right panel in plots.

**Figure 8:**
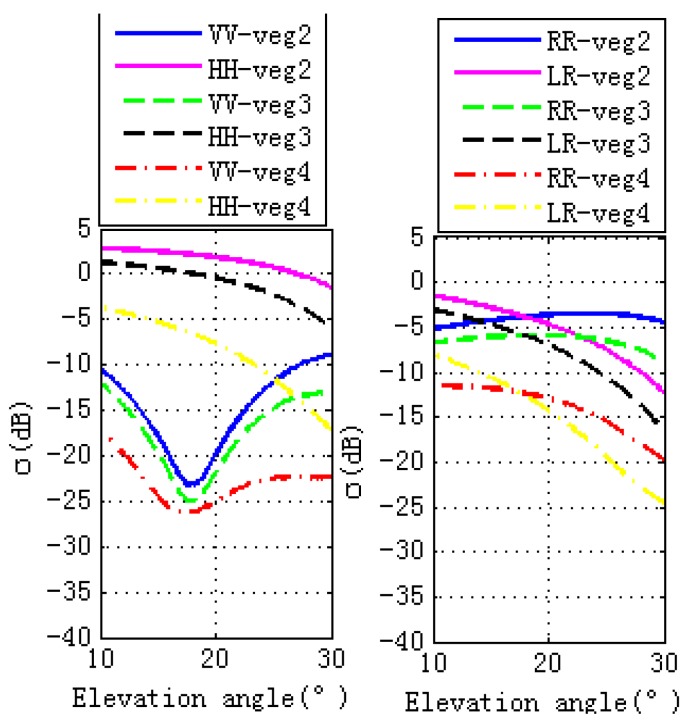
Specular scattering cross sections of different vegetation sizes. The left hand figure is for linear polarizations, while the right hand figure is for circular polarizations. Lengths and diameters of the stems or thicknesses and diameters of the leaves for veg 2, veg 3 and veg 4 are different. The sizes of veg 2 are smaller than veg 3, while the sizes of veg 4 are the largest.

During the vegetation growth period, stem and leaf sizes change. This section shows the effects of different stem and leaf lengths and diameters on GPS SNR, phase and code. Different wheat sizes, marked as Veg 2, Veg 3, and Veg 4, are shown in Table 3. The simulated GPS multipath observables are presented in the left panel of Figure 7, which illustrates that larger wheat sizes correspond to lower magnitude fluctuations of GPS multipath observables. The right hand panel of Figure 7 shows that larger vegetation size corresponds to lower peak amplitude of the GPS multipath observables. Changes in stem and leaf sizes corresponding to different scattering properties are shown in Figure 8. Due to vegetation attenuation, larger wheat sizes correspond to lower specular scattering cross sections (both linear and circular polarizations) and therefore they lead to the lower peak to peak magnitude fluctuations in the GPS multipath observables.

### 4.4. Ground Soil Moisture Effects

**Figure 9:**
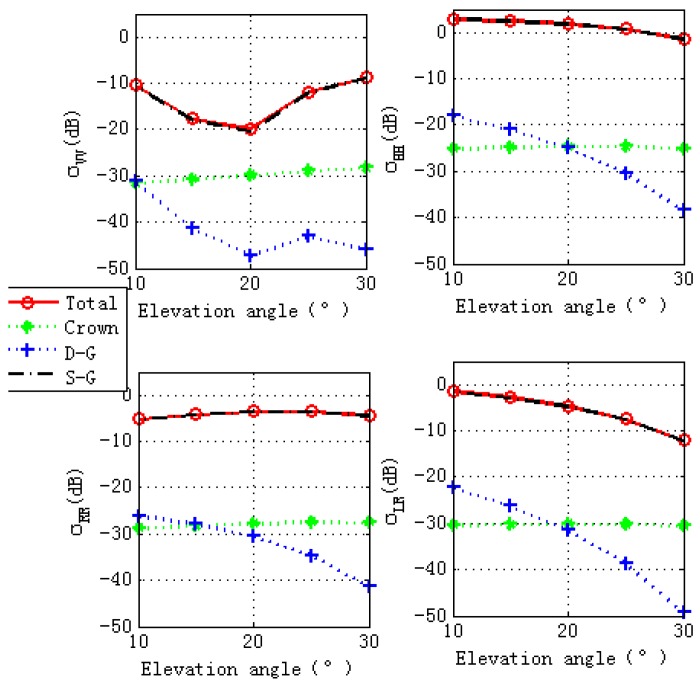
Vegetation scattering component contributions versus the specular incidence angles. The top panel is for linear polarization (VV and HH) and the bottom panel is for circular polarization (RR and LR). Total is the total scattering; Crown is the scattering from the crown layer; D-G is the Direct-ground scattering component; and S-G is the specular scattering component.

**Figure 10:**
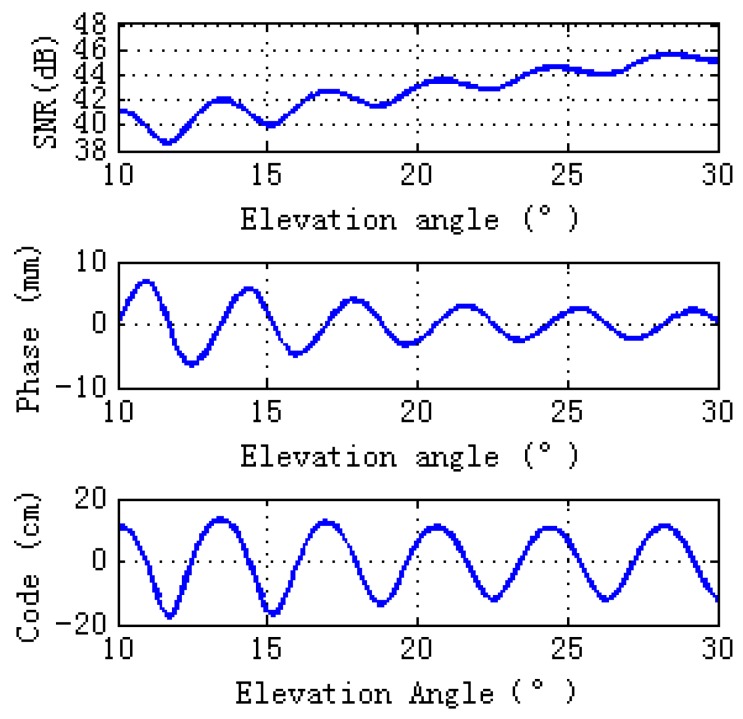
Effects of different ground soil moisture contents on the forward GPS multipath simulation. The ground soil moisture content for veg 2 is 0.15, while the one for veg 5 is 0.55.

**Figure 11:**
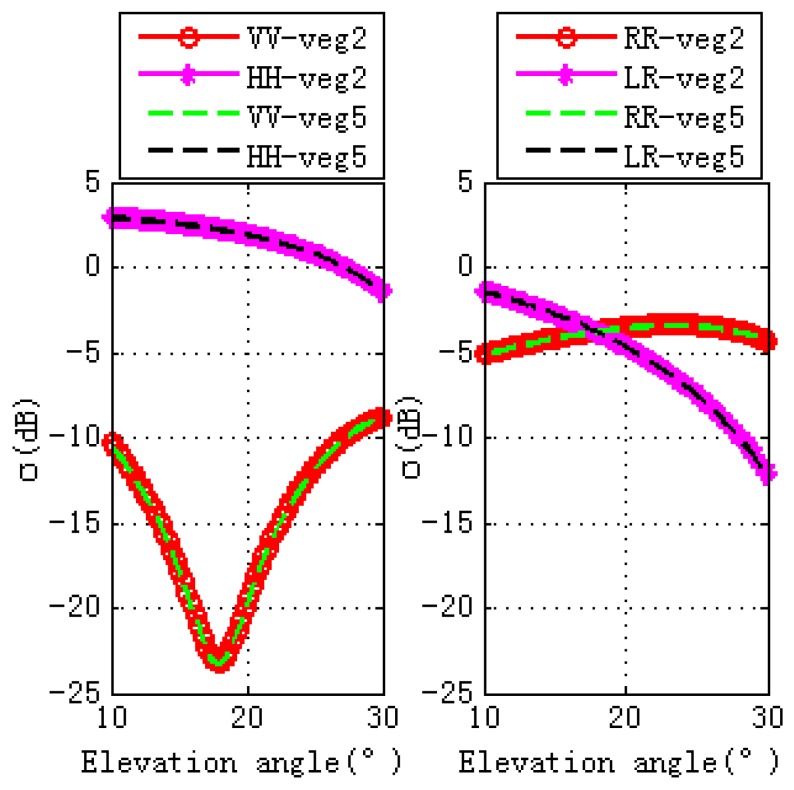
Specular scattering cross sections for Veg 2 and Veg 5. The ground soil moisture content for veg 2 is 0.15, while the one for veg 5 is 0.55. The left figure is for linear polarizations, while the right one is for circular polarizations.

**Figure 12:**
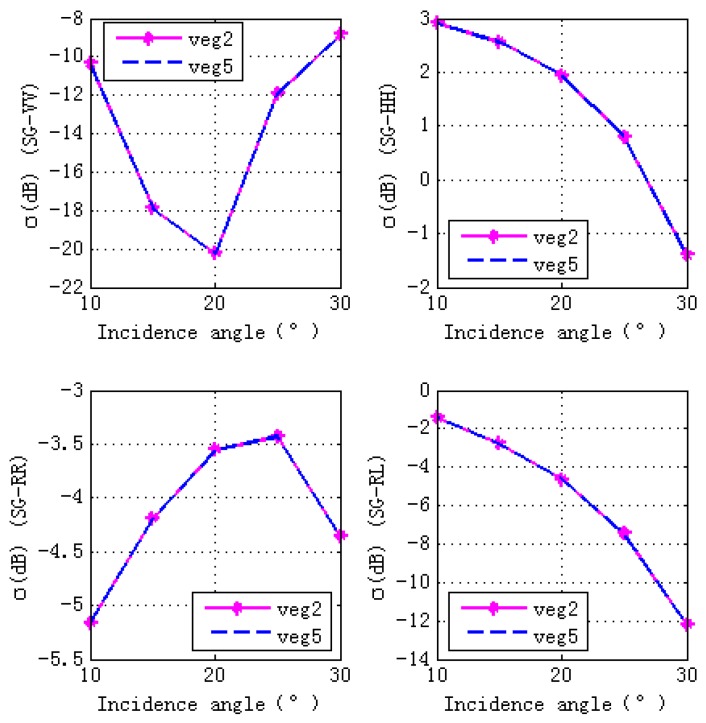
Scattering cross sections for S-G component with different ground soil moisture contents. The ground soil moisture content for veg 2 is 0.15, while the one for veg 5 is 0.55. The top panel is for linear polarization (VV and HH) and the bottom panel is for circular polarization (RR and LR).

Figure 9 shows contributions of different scattering components to the total scattering cross sections. The total scattering cross sections are dominated by S-G component. Therefore, we analyze the effects of ground soil moisture contents (shown in Table 4) on the final GPS multipath observables. It can be seen from Figure 10 that ground moisture content has almost no effects on final GPS multipath observables, the scattering properties illustrated by Figure 11 and Figure 12 give us the reasons: specular scattering cross sections for different ground soil moisture are almost the same, therefore vegetation covered ground soil moisture has almost no effects on the final GPS multipath observables.

## 5. Discussion

Once multipath signals were thought to be detrimental, but now they have been employed for geophysical parameter retrieval and have emerged as a new promising remote sensing techniques. In order to study the physical mechanisms of GPS-MR, a fully polarimetric forward GPS multipath simulator based on the Radiative Transfer Equation Model has been described in this paper. The developed model inserts a new element into the traditional GPS-MR simulator. In this way, we invite one kind of microwave scattering model into the forward GPS multipath model. The physical model is now able to describe the physical interaction processes between the scatters and electromagnetic waves. The microwave vegetation model can be divided into a continuous model and the discrete scattering model, which is often used for crop physical scattering models. The discrete scattering model is often further divided into an incoherence model and a coherence model. The coherence model considers the changes of phase and amplitude, while the incoherence model only considers the intensity changes. The model is based on the radiative transfer equation model, while the bistatic scattering MIMICS (Michigan Microwave Canopy Scattering Model) is a commonly used vegetation scattering model. The limitation of our newly developed model is that it only takes amplitude changes into consideration. In this way, we think that the incident GPS broadcast signals are superimposed on the direct signals, and their phase remains changed. The superposition of the reflected signals only results in the amplitude change of electromagnetic waves. It has been pointed out that phase is very sensitive to soil moisture. As shown in Chew et al. [12], the GPS multipath metric is an efficient method for the vegetation amount estimation, but phase is not a good indicator. Therefore, this newly developed model only takes the amplitude change of GPS broadcast signals into consideration.

## 6. Conclusions

GPS-MR is a new emerging remote sensing technique showing wide potential in soil moisture and vegetation detection. For data interpretation, sensitive analysis of the measured quantity to various interested parameters and vegetation parameters retrieval, theoretical models should be developed. Different from the original Fresnel reflection coefficients, we focus on the vegetation scattering properties on GPS multipath observables. For the first time, the first order radiative transfer equation model (Bi-mimics) is incorporated into the forward GPS multipath simulator. Effects of antenna type on the final SNR, phase and code are not considered here since we are focusing on the vegetation environment. Vegetation will affect the magnitude fluctuations of GPS multipath observables at different extents for different elevation angles. The simulation results show that larger vegetation moisture content and bigger vegetation sizes correspond to lower specular scattering cross sections and thus lead to lower magnitude fluctuations of the final GPS multipath signatures. Although the specular-ground component dominates the final scattering properties, ground soil moisture content has almost no effects on the final scattering cross sections and GPS multipath observables. From our simulations, it can be seen that GPS-MR is an efficient ground-based remote sensing technique for vegetation moisture content and growth detections. Using this model, it is more convenient to analyze the vegetation characteristics and it can explicitly link the GPS multipath signatures with the vegetation environment parameters. Our future work will concentrate on the development of algorithms for vegetation water content retrieval and the assessment of the potentialities of GPS-MR measurement for vegetation biomass monitoring.

## Figures and Tables

**Table 1:** Vegetation inputs for the simulator. Vegetation is modeled by a crown layer (composed of stem and leaf) and ground layer.

Stem		Leaf		Ground	
Moisture (gravimetric)	0.72	Moisture (gravimetric)	0.8	Soil RMS Height (cm)	0.45
Density (number/m^3^)	1000	Density (number/m^3^)	2500	Correlation length (cm)	18.75
Length (m)	0.35	Thickness (m)	0.02	Moisture (volumetric)	0.15
Diameter (cm)	0.3	Diameter (cm)	0.04	Soil % sand	10
Temperature (°C)	20	Temperature (°C)	20	Temperature (°C)	20
Distribution	Uniform	Distribution	Uniform	Soil % silt	60

**Table 2:** Different vegetation moisture contents. The stem and leaf moisture contents of vegetation 1 and vegetation 2 are different, while the other input parameters are the same.

**Vegetation 1**			
Stem		Leaf	
Moisture(gravimetric)	0.72	Moisture(gravimetric)	0.8
**Vegetation 2**			
Stem		Leaf	
Moisture(gravimetric)	0.2	Moisture(gravimetric)	0.2

**Table 3:** Different vegetation sizes as for Vegetation 2, Vegetation 3 and Vegetation 4. The length and diameter of stem and leaf are different, while the other input parameters are the same.

**Vegetation 2**			
Stem		Leaf	
length (m)	0.15	thickness (m)	0.01
diameter (cm)	0.3	diameter (cm)	0.02
**Vegetation 3**				
Stem		Leaf	
length (m)	0.35	thickness (m)	0.02
diameter (cm)	0.3	diameter (cm)	0.04
**Vegetation 4**			
Stem		Leaf	
length (m)	0.55	length (m)	0.04
diameter (cm)	0.4	diameter (cm)	0.05

**Table 4:** Different ground soil moisture for veg 2 and veg 5. As for Vegetation 2 and Vegetation 5, the ground soil moisture contents are different, while the other input parameters are the same.

	Ground (Volumetric Soil Moisture)
**Vegetation 2**	0.15
**Vegetation 5**	0.55

## Abbreviations

BAO-Tower	Boulder Atmospheric Observatory-tower
Bi-Mimics	Bistatic-Michigan Microwave Canopy Scattering Model
DBA	distorted Born approximation
DMR	Delay Doppler Maps Receiver
GNSS	Global Navigation Satellite System
GNSS-R	GNSS-Reflectometry
GPS	Global Positioning System
GPS-IR	GPS-Interferometric Reflectometry
GPS-MR	GPS multipath reflectometry
LEiMON	Land Monitoring with Navigation Signals
LHCP	Left Hand Circular Polarization
LR	The polarization of the transmitted signal is RHCP, while the received one is LHCP
Mimics	Michigan Microwave Canopy Scattering Model
PBO	plate boundary observatory
RHCP	Right Hand Circular Polarization
RR	The polarization of the transmitted signal is RHCP, while the received one is also RHCP
RT	radiative transfer model
SMEX	Soil Moisture Experiments
SMIGOL	Soil Moisture Interference pattern GNSS Observations at L-band Reflectometer
SNR	Signal-to Noise Ratio
Spec-Mimics	Specular-Michigan Microwave Canopy Scattering Model
